# Awareness and utilization of Swedish youth clinics among migrants enrolled in Swedish language programmes: a cross-sectional study

**DOI:** 10.1080/16549716.2024.2401658

**Published:** 2024-09-11

**Authors:** Amanda Lundberg, Anna-Karin Hurtig, Faustine Kyungu Nkulu-Kalengayi

**Affiliations:** Department of Epidemiology and Global Health, Umeå University, Umeå, Sweden

**Keywords:** Youths/young adults, immigrants, adolescent health services, survey, access to health services, healthcare disparities, Sweden

## Abstract

**Background:**

Previous research has highlighted inequalities in access to Swedish youth clinics (YCs). These inequalities exist not only between non-migrant and young migrant populations but also within various migrant groups.

**Objectives:**

To assess awareness and utilization of Swedish YCs among migrants enrolled in Swedish language programmes and explore associated factors.

**Methods:**

This cross-sectional study involved 1,112 migrants aged 15−65. The analytical sample included 642 (57%) participants who answered the main outcome question about awareness of YCs. Descriptive statistics, bivariate, and multivariate log-binomial regression analyses using a Bayesian approach were applied to summarize the data and identify factors associated with awareness and utilization of YCs among migrants.

**Results:**

The results revealed that 30% of all participants and 40% of the participants aged 15–25 years had heard of YCs. Additionally, 23% of the target group (15−25 years) had ever visited one. During descriptive and bivariate analyses, socio-demographic variations were evident in YCs’ awareness and utilization. However, in multivariate analyses, only the associations between awareness and year of arrival, and YCs’ utilization and year of arrival and type of residence permit, remained statistically significant.

**Conclusion:**

This study highlights the level of awareness of YCs among migrants attending Swedish language programmes and their utilization by those aged 15−25 years, potentially impacting their access to crucial services and resources. Targeted interventions and sustainable strategies beyond one-time interventions are essential to address the specific needs of different socio-demographic groups and ensure equitable access to YCs’ information and services.

## Paper context

- **Main findings**: This study highlights the level of awareness and utilization of youth clinics among migrants attending Swedish language programmes, revealing socio-demographic disparities across different groups.
- **Added knowledge**: Migrant men and unaccompanied minors are more likely to be aware of and use youth clinics than migrant women and those with legal guardians, while recent migrants and those with temporary residence permits are at an increased risk of being unaware of or not utilizing these services.
- **Global health impact for policy and action**: Targeted interventions and sustainable strategies, beyond one-time efforts, are essential to address the specific needs of different migrant subgroups and ensure equitable and universal access to crucial information and services related to sexual and reproductive health and rights.

## Background

Youth clinics (YCs) constitute a nationwide network of health services for young people in Sweden, fully integrated into the public healthcare system. Their primary focus is addressing the health needs of young people, particularly those related to sexual and reproductive health (SRH). The YCs have been operational for over five decades and currently provide services through a multi-disciplinary team, with a special focus on sexual and reproductive health and mental well-being [1]. There are approximately 300 YCs available to young people aged 12−25 years. However, age limits vary between regions, clinics, and services. Importantly, the services are perceived as a fundamental component of the Swedish healthcare system, and all young people are introduced to them in school [2]. YCs offer information and services to young people free of charge extending their accessibility to all, including young asylum seekers and undocumented migrants (≤18 years) [1]. Young people can access information and services offered by YCs online by visiting the Youth Clinic Online (*Ungdomsmottagning på nätet*: UMO) website. The website is a complement to the physical YCs. It targets young people aged 13−25 and provides information about the body, sex, relationships, mental health, alcohol and drugs, self-esteem, and much more. However, since the site is only available in Swedish, the Youmo website (Youmo.se) has been created to make this information accessible to young people with limited fluency in Swedish. Youmo.se focuses on sex, health, and relationships for young people aged 13−20 who are new to Sweden. It also provides methodological resources for professionals working with this target group, as well as training on how to discuss the topics covered on Youmo.se with young people. To ensure accessibility for young migrants, Youmo offers parts of UMO’s content in Arabic, Dari, English, Somali, simple Swedish, and Tigrinya [3,4]. YCs play a crucial role in sustaining, promoting, and raising awareness about sexual and reproductive health and rights (SRHR) among youths in Sweden. Young people often seek out YCs independently. They may also be referred by their school or other healthcare providers. Occasionally, a parent initiates the contact. YCs’ approach involves assessing the young person’s overall situation regardless of the initial reason for their visit [5].

However, it is noteworthy that young people in Sweden encompass a diverse group of people with various ethnic, cultural, religious, and linguistic backgrounds, largely due to immigration over the last decades. Migrant youths are a rapidly growing demographic group [6].

Nevertheless, there is no generally accepted definition of the term ‘migrant’. In this study, we adopted the International Organization for Migration definition, which defines international migrants as people who move away from their countries of usual residence temporarily or permanently, and for a variety of reasons to settle in another country [7]. However, the term includes different categories of people from different countries, with different socio-cultural backgrounds, legal statuses, duration of stay, and ages resulting in different needs and different ability to access available services. Statistics Sweden defines (im)migrants (*Invandrare*) as all individuals who move to Sweden and are registered in the Swedish population register. To be registered in the population register, one must have the intention and right to stay in Sweden for at least 12 months [8]. The rules vary for different groups of migrants. For instance, Nordic citizens can freely immigrate to Sweden, while EU citizens outside the Nordic countries need to apply for the right of residence (*uppehållsrätt*) to stay in the country for more than 3 months. Citizens of other countries need a residence permit issued by the Swedish Migration Agency [8]. As of 2021, approximately one-fifth of the Swedish population was foreign-born [9] and foreign-born youths represented roughly 19% of the population aged 12−25 years in Sweden [6]. In this study, young migrants are defined as foreign-born young people aged 15−25 years regardless of their legal status, reason for migration and time spent in Sweden.

One of the key targets of Goal 5 of the 2030 Agenda for Sustainable Development is ensuring universal access to SRHR to attain sexual and reproductive health equity [10]. This commitment requires the fulfilment, respect, and protection of the right of all individuals to access SRH-related information, resources, services, and support throughout their lives, free from discrimination, coercion, exploitation, and violence regardless of their background [11]. However, it is important to acknowledge that YCs are not equally accessible to all young people residing in Sweden. Previous research has indicated disparities in access to YCs, not only between non-migrant and young migrant populations but also within various migrant groups [2,12–14].

Both young age and migration experience increase the risk and vulnerability to poor SRH [15–17]. The Swedish SRHR plan of action recognizes young people and people with migration experience as priority groups [18]. Nevertheless, evidence suggests that young migrants, despite their high healthcare needs, encounter barriers to accessing available services in their receiving countries, particularly those relating to SRHR, including those provided by YCs [15,16,19]. These barriers include language, legal (i.e. asylum seekers and undocumented migrants over 18 years are only entitled to care that cannot be postponed) [20] and cultural obstacles, limited health literacy and inadequate knowledge about available services and rights, and a lack of familiarity with the Swedish health system [2,12,13]. Furthermore, research has highlighted that newcomers often lack access to knowledge and information about SRHR. Typically, Swedish for Immigrants (SFI) programmes, adult education, and language introduction classes do not adequately provide sex and relationship education. Additionally, young newcomers may not be well-informed about relevant societal functions and methods of independently acquiring information related to SRH [21]. As a result, since 2018, Regulation 2017:829 mandates civic orientation (CO) for certain newly arrived migrants in each municipality according to the law (2013:156). This programme aims to facilitate their integration into work and society. The county administrative board oversees the programme, and municipalities ensure recent migrants receive it. The programme covers topics such as Swedish society, human rights, democratic values, and individual responsibilities; it includes modules about coming to Sweden, life in Sweden, self-sufficiency and personal development, family life and child-rearing, societal involvement, ageing, and how to care for one’s health, which includes information about health and how the Swedish health system works. CO should start promptly after an establishment plan is in place, ideally completed within a year, and consists of at least 100 hours. It can be combined with work, studies, and other activities. Whenever possible, it should be conducted in the participant’s native language, a proficient language, or in Swedish with an interpreter [22].

Despite the availability of YCs and a National Strategy for SRHR, studies have revealed limitations and disparities in young people’s access to SRHR-related information and services, including YCs [23]. The 2015 report by the Swedish Agency for Youth and Civil Society (MUCF) highlighted that young migrants in Sweden face more challenges or barriers when it comes to accessing services and information related to their SRH compared to other young people in the country [21,24]. A national survey of YCs conducted by Sweden’s Municipalities and Regions *(Sveriges Kommuner och Regioner: SKR*) has highlighted the need to reach out to boys to a greater extent and to target young people in socio-economically vulnerable situations, including young migrants [25]. Additionally, a Swedish survey of migrants’ SRHR has indicated that young migrants report poorer general and sexual health more frequently but visit YCs less often than their native-born peers [15]. Nevertheless, the SKR report stressed that the barriers faced by young people who do not utilize YCs can vary across regions and among various groups of young people [25]. This study is part of a broader project that seeks to explore how and under what circumstances Swedish YCs are reaching out to young migrants. The study aims to assess and describe the awareness and utilization of Swedish YCs among migrant students enrolled in Swedish language programmes and explore associated factors.

## Methods

### Study design and setting

This cross-sectional survey was conducted among participants in SFI, municipal adult education (*Komvux*) and Language Introduction programmes at upper secondary schools (*Gymnasieskolan*). The study used a stratified sample (referred to as ‘mini-Sweden’) comprising 30 municipalities spread throughout Sweden from northern Övertorneå to the Southern Karlshamn Municipality.

SFI provides basic Swedish language education for migrants over 16, aiming to facilitate communication in daily life, civic contexts, work, and studies. The programme also offers literacy training for those unable to read or write in their native language [26]. SFI is tailored to individuals’ needs with personalized study plans, allowing students to progress at their own pace. It offers three study tracks. Track 1 is for students with little or no school background and includes courses A, B, C, and D. Track 2 is designed for students with less schooling than Swedish upper-secondary level; it covers courses B, C, and D. Track 3 is intended for students with education equivalent to Swedish upper secondary or university level, offering courses C and D. Students receive at least 15 hours of instruction per week. Courses can be taken at municipal or folk high schools and combined with work, placements, or other educational programmes like CO [26]. After SFI courses, students can move on to taking Basic Swedish as a second language (*Grundläggande svenska som andraspråk*) at municipal adult education (*Komvux*), which grants them access to more adult education towards a profession or higher education [27].

Language Introduction is an introductory programme for recent migrants aged 16–19 who do not have passing grades for national programmes. It focuses on developing Swedish language skills, allowing students to transition to other introductory programmes, national programmes, adult education, or professions. The education is tailored to each student’s experiences, knowledge, and educational goals [28].

### Study population

The knowledge and perspectives of migrant communities particularly parents of young migrants and the young migrants themselves significantly impact young migrants’ health-seeking behaviour and access to SRH services [29–31]. Thus, the target population for this study consisted of migrants aged 16−65 years attending Swedish language programmes. The inclusion criteria were being a migrant aged 16−65 years and being enrolled in SFI schools, a Language Introduction programme at upper secondary (*Gymnasieskolan*) schools or Swedish as a Second Language (SAS) at Komvux.

### Data collection

The survey was conducted by a private company, Invandrarindex, that has been hired since 2014 by authorities, municipalities, organizations/universities, and researchers to collect data yearly on newly arrived migrants’ perceptions about Swedish society [32]. The 2020 survey was administered via a web survey that was answered in classrooms, or in some cases digitally at home, during teacher-led lessons at SFI and the Language Introduction programme at upper secondary schools. Contrary to previous years, the survey could not be administered face-to-face as many schools were closed due to the COVID-19 pandemic. The survey questionnaire was available in six different languages: Arabic, English, Farsi, Somali, Swedish, and Tigrinya. A convenience sample of 1,122 migrants participated in the 2020 Immigrant Index (*Invandrarindex*) survey. Among the 656 (59%) respondents who mentioned their educational programmes, 69% were attending SFI schools, 22% were enrolled in Language Introduction programmes at upper secondary schools, and 9% were pursuing municipal adult education (Komvux) after completing SFI.

### Measures

The survey regarding YCs consisted of 12 socio-demographic questions and 7 specific questions about knowledge and utilization of YCs based on existing literature [2,14,29]. The answers from the survey were entered into Stata statistical software version 14.2.

#### Dependent variables

In this study, 2 of the 12 questions were used as outcome variables: ‘Have you ever heard about youth clinics?’ and ‘Have you ever visited a youth clinic?’ They were dichotomized as: ‘yes’ and ‘no’.

#### Dependent variables

The explanatory or independent variables included the following. (i) Age was compiled into three categories, 15–25 years (‘youths’), 26–40 years (‘young and middle-aged adults’), and above 40 years (‘old adults’). (ii) For practical reasons, participants’ countries of origin were gathered into six different world regions based on the Sustainable Development Goals (SDGs) regional grouping [33], these being Europe, the Middle East, and North Africa (MENA), sub-Saharan Africa (SSA), America, Asia, and the Pacific and ‘other’ for those who did not answer or provide irrelevant answers. (iii) Educational level was recoded into four categories depending on the number of years spent in school before migration (0 −6, 7−9, 10−12, and 13 years or more). (iv) Time spent in Sweden or time since arrival was compiled into three categories: 2019− 2020 (less than 2 years), 2017−2018 (2−3 years), and 2016 or earlier (4 years or more). (v) The reason for migration was categorized as follows: need for protection (quota refugees and asylum seekers), family ties, and work or studies. (vi) Type of residence permit was categorized as follows: permanent, temporary, uncertain − which included those awaiting a decision (‘I don’t know yet’) − and other (‘I don’t know’ or ‘I don’t want to answer’). (vii) Participants’ occupations were compiled into three categories: working, studying, or other. (viii) Place of residence in Sweden was grouped into three main geographical areas based on/adapted to the National Health Competence Council regional grouping as (1) the northern region, (2) the Stockholm and central Sweden region, and (3) the south, west, and southeast regions [34]. (ix) Religion was categorized as Christianity, Islam, other (Hinduism, Buddhism, and others), and atheism.

#### Other variable

Participants who were aware of YCs also answered a question about their sources of information: ‘Where did you get information about YCs?’ The answer options were as follows: sex education in schools, school nurse or school counsellor/social worker, parent, friend, UMO, Youmo, or other sources.

## Data analysis

The analytical sample for the outcome ‘awareness’, included 642 (57%) of the total 1, 122 survey participants who answered the question: ‘Have you ever heard about youth clinics?’. Among them, 40% (*n* = 256) chose to answer the questionnaire in Swedish, 34% (*n* = 217), in Arabic, 11% (*n* = 70) in English, 6% (*n* = 40) in Farsi, and another 6% (*n* = 41) in Tigrinya. The remaining 3% (*n* = 18) responded in Somali. For the outcome ‘utilization’, a sub-sample of 221 young participants (aged 25 years or younger) who responded positively to the question: ‘Have you ever visited a youth clinic?’ was selected. This age group was included since they are the primary target audience for YCs [1]. Descriptive analyses were performed to summarize data. All variable categories achieving less than 5% were either merged (e.g. for the variable region of birth, North, Central, and South America were merged with ‘other’) or considered as missing data (e.g. for the gender variable, the categories ‘other’ and ‘don’t want to answer’). Thereafter, a Bayesian approach to the regression analyses was applied since this method is better equipped to model data with small sample sizes. Log-binomial regression analyses were performed to estimate prevalence ratios as a measure of association and their 95% credible intervals for inferential purposes; in this way, uncertainty could be expressed as the 95% probability that the true (unknown) estimate would lie within the interval, given the evidence provided by the observed data. Univariable (unadjusted) models were calculated first followed by multivariable models where statistically significant variables in the former were included. As is common in the literature, we used a Student *t*-distribution with 7 degrees of freedom and a scale of 2.5 for the priors, since this is considered a reasonable default prior when coefficients should be close to 0 but have some chance of being large [35].

## Results

### The characteristics of participants

Approximately two-thirds (61%) of the 642 participants identified themselves as women, and one-third (35%) as men. Roughly one-third of the participants (35%) were 25 years old or younger at the time of the survey, 39% were 26–40, and 26% were over 40 years old. More than half (54%) grew up in the MENA region. Nearly half moved for family reunion (47%) and 4 in 10 sought asylum (41%). More than half identified as Muslim (53%), while Christians and Jews made up 22%. These sample characteristics are presented in Table 1.

**Table 1.** The socio-demographic characteristics of participants (*N* = 642).

Variables	Number (%)
**Gender**	
Men	223 (34.7)
Women	394 (61.3)
Other	5 (0.8)
Don’t want to answer	20 (3.1)
**Living with children**	
Yes	373 (58.1)
No	269 (41.9)
**Age (years)**	
25 or younger	221 (34.4)
26–40	248 (38.6)
Over 40	163 (25.4)
Missing	10 (1.6)
**Region of origin**	
Europe	78 (11.8)
Middle East and North Africa	340 (53.0)
Sub-Saharan Africa	85 (13.2)
North, South, and Central America	13 (2.0)
Asia and the Pacific	99 (15.4)
Other	16 (2.5)
Missing	11 (1.7)
**Prior education (years)**	
0–6	186 (29.0)
7–9	152 (23.6)
10–12	139 (21.7)
13 or more	152 (23.8)
Missing	13 (2.0)
**Year of arrival in Sweden**	
2019–2020	192 (30.0)
2017–2018	204 (31.8)
2016 or earlier Missing	240 (37.4) 6 (0.9)
**Reason for migration**	
Quota refugee or asylum seeker	259 (40.3)
Family reunion	295 (46.0)
Work or studies	41 (6.4)
I don’t understand the question	37 (5.8)
Missing	10 (1.6)
**Type of residence permit**	
Permanent	364 (56.7)
Temporary	196 (30.5)
Uncertain (I don’t know yet, I don’t want to answer, or I don’t know)	56 (8.7)
Missing	26 (4.0)
**Arrived as unaccompanied minor**	
Yes	72 (11.2)
No	539 (84.0)
Missing/NA	31 (4.8)
**Region of residence in Sweden**	
The Northern region	70 (10.9)
Uppsala–Örebro or Stockholm’s region	271 (42.2)
The southern, western, or southeastern region	301 (47.0)
**Main occupation**	
Work	59 (9.2)
Studies	493 (76.8)
Other	90 (14.0)
**Religion**	
Atheism	94 (14.6)
Christianity and Judaism	120 (18.7)
Islam	287 (44.7)
Other religion Missing	39 (6.1) 102 (15.9)

### To what extent are migrants aware of youth clinics?

Overall, only 3 in 10 participants (30%) had ever heard of YCs. Participants who identified themselves as men (35%), those aged 15−25 years (40%), those who had 13 or more years of education (20%), those who arrived in Sweden in 2017−2018 (35%), those who originated from the SSA (44.%) region, those who migrate to seek protection (35%) or as unaccompanied minors (49%), and those who were Christians (42%) reported being aware of YCs to a greater extent than their counterparts (see Table 2). The sources of information about YCs were reported in the following order: school nurse or school counsellor/social worker (39%), friends (32%), other sources (17%), sex education in schools (14%), parents (14%), UMO (9%), and Youmo (7%).

**Table 2.** Prevalence of awareness of youth clinics and crude and adjusted prevalence ratios with their corresponding 95% credible intervals (CrI).

Variables	Prevalence n (%)	Crude PR (95% CrI)	Adjusted PR (95% CrI)
**Gender**			
Women	110 (27.9)	1	
Men	77 (34.5)	1.24 (0.97, 1.54)	
**Living with children**			
Yes	112 (30.0)	1	
No	82 (30.5)	1.02 (0.79, 1.29)	
**Age group (years)**			
>40	41 (25.2)	1	1
26–40	60 (24.2)	0.96 (0.69, 1.35)	0.94 (0.67, 1.37)
15–25	88 (39.8)	**1.59 (1.17, 2.18**)	1.24 (0.87, 1.78)
**Country of origin/home country**			
Europe	18 (32.7)	1	
Middle East and North Africa	70 (23.8)	0.74 (0.50, 1.18)	
Sub-Saharan Africa	27 (44.7)	1.46 (0.94, 2.38)	
Other	76 (33.8)	1.05 (0.72, 1.65)	
**Prior education (years)**			
0–6	60 (32.3)	1	1
7–9	48 (31.6)	0.97 (0.70, 1.34)	1.15 (0.83, 1.61)
10–12	52 (37.4)	1.16 (0.86, 1.56)	1.36 (0.98, 1.92)
13 or more	31 (20.4)	**0.63 (0.42, 0.90)**	0.85 (0.55, 1.27)
**Year of arrival**			
2019–2020	47 (24.5)	1	1
2017–2018	71 (34.8)	**1.42 (1.04, 1.94)**	**1.43 (1.05, 2.00)**
2016 or earlier	74 (30.8)	1.25 (0.91, 1.72)	1.24 (0.89, 1.78)
**Reason for migration**			
Quota refugee or asylum seeker	91 (35.1)	1	1
Family reunion	76 (25.8)	**0.73 (0.57, 0.95)**	0.82 (0.62, 1.08)
Work or study	10 (24.4)	0.66 (0.35, 1.11)	0.77 (0.41, 1.23)
I don’t understand the question	12 (32.4)	0.89 (0.51, 1.37)	0.99 (0.55, 1.53)
**Type of residence permit**			
Permanent	115 (31.6)	1	
Temporary	49 (25.0)	0.79 (0.59, 1.05)	
I don’t know yet/I don’t want to answer/I don’t know	21 (37.5)	1.17 (0.78, 1.64)	
**Arrived as unaccompanied minor**			
No	146 (27.1)	1	1
Yes	35 (48.6)	**1.79 (1.33, 2.32**)	1.30 (0.93, 1.75)
**Region of residence in Sweden**			
Northern	22 (31.4)	1	
Uppsala–Örebro–Stockholm	72 (26.6)	0.84 (0.59, 1.33)	
Southern–western–southeastern	100 (33.2)	1.06 (0.76, 1.65)	
**Main occupation**			
Work	23 (39.0)	1	1
Studies	150 (30.4)	0.80 (0.58, 1.15)	0.86 (0.61, 1.26)
Other	21 (23.3)	**0.60 (0.37, 0.96)**	0.78 (0.46, 1.30)
**Religion**			
Atheism	31 (33.0)	1	
Christianity	50 (42.0)	1.27 (0.91, 1.85)	
Islam	71 (24.7)	0.76 (0.54, 1.09)	
Other religion	10 (25.0)	0.75 (0.38, 1.35)	

Note: Significant results are highlighted in bold (p < .05).

### Factors associated with awareness of youth clinics

The bivariate analyses revealed associations between certain socio-demographic variables and awareness of YCs. Younger migrants (15−25 years), those who arrived in Sweden between 2017 and 2018, and those who arrived as unaccompanied minors had a higher prevalence of awareness than the reference groups. Conversely, those with higher/at least tertiary education prior to migration (≥13 years), those who moved for family reunion, and those who had an occupation other than work or studies had lower prevalence of awareness of YCs (were more likely to be unaware) than their counterparts. However, only the association between year of arrival and awareness of YCs remained statistically significant in the multivariable analysis after adjusting for potential confounders (see Table 2).

### To what extent have migrant youths visited youth clinics?

Of the 221 young participants (15−25 years) who answered the question about utilization, barely 23% stated that they had visited a YC. Young men (28%), participants from SSA (36%), those who arrived in Sweden in 2016 or earlier (37%), those with prior school attendance of 6 years or less (32%), and those who arrived as asylum seekers/quota refugees (28%) or unaccompanied minors (39%) visited the YCs to a greater extent than other groups. Muslims were the group with the lowest prevalence (18%) of those who had visited a YC (see Table 3).

**Table 3.** Prevalence of utilization of youth clinics and crude and adjusted prevalence ratios with their corresponding 95% credible intervals (CrI).

Variables	Prevalence n (%)	Crude PR (95% CrI)	Adjusted PR (95% CrI)
**Gender**			
Young women	21 (18.9)	1	
Young men	28 (27.7)	1.48 (0.90, 2.45)	
**Living with children**			
Yes	19 (24.7)	1	
No	31 (21.5)	0.86 (0.53, 1.46)	
**Region of origin**			
Europe	2 (15.4)	1	
Middle East and North Africa	24 (19.5)	1.30 (0.49, 5.62)	
Sub-Saharan Africa	5 (35.7)	2.22 (0.67, 9.83)	
Other	17 (25.4)	1.65 (0.63, 7.13)	
**Prior education (years)**			
0–6	28 (31.8)	1	1
7–9	8 (15.4)	0.48 (0.22, 0.92)	0.56 (0.25, 1.07)
10–12	9 (16.4)	0.51 (0.25, 0.95)	0.88 (0.43, 1.63)
13 or more	5 (21.7)	0.64 (0.24, 1.32)	0.90 (0.29, 1.81)
**Year of arrival**			
2019–2020	7 (9.6)	1	1
2017–2018	21 (23.3)	**2.36 (1.12, 5.61)**	**2.13 (0.98, 5.11)**
2016 or earlier	21 (36.8)	**3.81 (1.83, 8.70)**	**3.09 (1.44, 7.39)**
**Reason for migration**			
Quota refugee or asylum seeker	27 (28.4)	1	1
Family reunion	14 (16.1)	**0.57 (0.32, 1.00)**	0.70 (0.37, 1.18)
Work or study	2 (15.4)	0.50 (0.10, 1.41)	0.60 (0.10, 1.64)
I don’t understand the question	6 (28.6)	0.97 (0.40, 1.92)	0.87 (0.35, 1.60)
**Type of residence permit**			
Permanent	33 (26.8)	1	1
Temporary	8 (13.1)	**0.48 (0.22, 0.90)**	**0.51 (0.23, 1.00)**
I don’t know yet/I don’t want to answer/I don’t know	6 (20.0)	0.72 (0.28, 1.39)	0.88 (0.38, 1.63)
**Arrived as unaccompanied minor**			
No	31 (18.7)	1	1
Yes	18 (39.1)	**2.03 (1.24, 3.33)**	1.61 (0.98, 2.58)
**Region of residence in Sweden**			
Northern	3 (18.8)	1	
Uppsala–Örebro–Stockholm	12 (16.9)	0.97 (0.35, 3.20)	
Southern–western–southeastern	35 (26.1)	1.54 (0.64, 4.88)	
**Main occupation**			
Work	6 (37.5)	1	
Studies	42 (21.8)	0.63 (0.35, 1.44)	
Other	2 (16.7)	0.44 (0.08, 1.60)	
**Religion**			
Atheism	7 (25.0)	1	
Christianity	11 (35.5)	1.43 (0.67, 3.28)	
Islam	22 (18.2)	0.75 (0.38, 1.72)	
Other religion	3 (30.0)	1.08 (0.30, 3.14)	

Note: Significant results are highlighted in bold (p < .05).

### Factors associated with utilization of youth clinics

The bivariate analysis results show associations between certain socio-demographic variables and utilization of YCs among young migrants. Young migrants who arrived before 2019 and those who moved as unaccompanied minors had higher prevalence of YC utilization than their counterparts. Conversely, those with 7−12 years of education prior to migration, those who migrated for family reunion, and those with a temporary residence permit were more likely to have lower prevalence of utilization of a YC compared to their counterparts. Most of these associations did not remain statistically significant in the multivariate analyses, except for the variables: year of arrival and type of residence permit (see Table 3).

## Discussion

This study shows that only 30% of all participants and 40% of the young participants aged 15−25 years had heard about YCs. Additionally, 23% of the target group (15−25 years) had ever visited one. Nevertheless, there are noteworthy disparities in both awareness and utilization of YCs across socio-demographic groups, based on factors such as age, gender, educational background, geographic origin, religion, reason for migration, duration of stay, type of migration, and age at migration. The bivariate analyses revealed associations between certain socio-demographic factors and awareness or utilization of YCs, including age, education, year of arrival, reason for migration, type of residence permit (only for utilization), and whether they migrated as unaccompanied minors. However, in the multivariate analyses, only year of arrival showed a statistically significant association with awareness of YCs. For utilization of YCs, statistically significant associations remained with year of arrival and type of residence permit.

The study reveals a limited awareness of YCs among participants. Only 30% of participants overall and 40% of the young participants aged 15−25 years reported having heard about YCs. This indicates a substantial lack of awareness regarding the existence and function of YCs among migrants enrolled in Swedish language programmes. It is worth noting that YCs are vital for promoting and fulfilling young people’s SRHR. However, these services are only accessible to those who are aware of their existence and can reach them [2]. In a previous study, young migrants commonly reported refraining from seeking sexual and reproductive healthcare (SRHC) due to a lack of knowledge about the Swedish health system and available SRHC services [13]. Thus, the limited utilization rate of YCs (23%) found in this study can be attributed partly to the limited awareness reported here and in other studies [2,12,13,15,36]. In another Swedish study on young migrants’ SRHR, approximately 40% of the respondents were unaware of where to access information about sexuality and sexual health [15]. Knowledge of the availability and function of YCs is essential for informed decision-making and access to the services and resources they provide. This indicates that the information on youth-friendly clinics and sexual health aimed at young people aged 13−25, available in Swedish and five other foreign languages (Arabic, Dari, English, Somali, and Tigrinya) on national youth guidance centre websites UMO (9%) and Youmo (7%), is not readily accessible to young migrants [3,4]. A 2019 brand survey of young people aged 15−25 revealed that 87% were aware of UMO [37]. Among participants aware of YCs, the most commonly mentioned sources of information were the school nurse or school counsellor/social worker (39%) and friends (32%). However, the utilization rate was slightly higher than the 20% reported in a previous study. That study showed that among young and young adult migrants (16−29 years) who accessed SRHC services in the past 12 months, the most visited healthcare facilities were primary care centres (43%), youth clinics (20%), women’s clinics (16%), and maternal health centres (15%) [38]. The discrepancy in utilization rates might be attributed to the different age groups of the samples in the two studies.

Likewise, the socio-demographic inequalities in awareness and utilization of YCs, as observed in the descriptive and bivariate analyses of this study, merit careful consideration. Previous studies have highlighted similar disparities in knowledge and utilization of SRHC services among migrants [13,38,39]. These disparities may arise from the heterogeneity of migrants with regard to age, reasons for migration, socioeconomic conditions, time since migration, and cultural and religious backgrounds, leading to diverse needs, knowledge levels, and ability to use the available services [13,15,16]. For instance, recent migrants may face more challenges accessing information and available services due to unfamiliarity with the system, a language barrier or legal status [21,36]. It is understandable that age and educational-level differences play a role in awareness and utilization of YCs, as YCs typically target people under 25 years of age, and a person’s level of education can significantly impact awareness [13]. A previous study showed that young adults aged 26−29 years visited primary care centres more frequently (60%), whereas teenagers aged 16–19 years were more likely to visit YCs (46%) [38]. Nevertheless, it is crucial to address myths and misconceptions about YCs revealed in other studies that may discourage young migrants from utilizing YCs [29,36]. Our results further indicate that participants from SSA and other countries exhibited the highest awareness prevalence. Additionally, young people in these regions utilized YCs more frequently than their counterparts in Europe and the MENA regions. This disparity may be attributed to the fact that migrants from high HIV prevalence areas, primarily SSA, are targeted by HIV prevention programmes that also provide information about available HIV and STI testing and treatment services available at YCs.

Contrary to findings from previous Swedish studies on young people’s access to YCs [2,13,15], our descriptive and bivariate results reveal that young migrant men and boys exhibit higher awareness and visit YCs more frequently than migrant women and girls. This pattern was also observed in another study that showed that the proportion of migrant boys among those who visited the YC (27%) was twice as high compared to migrant girls (13%) [38]. Previous research suggests that this discrepancy may stem from gender-based cultural and religious norms surrounding young people’s sexuality. These norms can impact young migrant women and girls’ access to SRH-related information and services [16,30,31]. The stigma and taboo surrounding issues like pre-marital sex and sexually transmitted infections (STIs) in certain cultures and religions can act as barriers to young migrant women and girls’ access to SRH-related information and services [31]. Socio-cultural backgrounds, particularly those of parents, have been found to influence perceptions of YCs and the seeking behaviour of young migrant women and girls concerning SRH services [30,40]. Moreover, migrant parents have expressed the desire for YCs to acknowledge their cultural backgrounds, norms, and beliefs while providing SRH services to their children [29]. Engaging with migrant parents and communities can help destigmatize sexual health problems, including STIs and premarital sex [30]. Last but not least, unaccompanied minors and asylum seekers have been the target of various interventions aimed at promoting social integration in Sweden, which may partly explain why those who moved as unaccompanied minors or to seek protection were more aware of and used YCs to a greater extent than their peers who moved with family members [41]. This could partly account for the higher proportion of boys in this sample who had visited or were aware of YCs, as the majority of unaccompanied minors are boys: for example, in 2015, Sweden saw an unprecedented influx of approximately 35,000 unaccompanied minors seeking protection, boys making up 92% of this group. In other years, the percentage of boys among unaccompanied minors has ranged from 58% to 85% [42].

However, in multivariate analyses, only the associations between year of arrival and awareness, and between utilization of YCs and both year of arrival and type of residence, remained statistically significant. The high prevalence of awareness observed among those who arrived between 2017 and 2018 may indicate the positive impact of short-term integration programmess initiated after the 2015 migration from Arab Spring countries to Sweden. These programmes provided migrants with information about Swedish society, including the healthcare system. However, these programmes may no longer be available for recent migrants or did not exist before [12]. Additionally, barriers to accessing information and services may persist among recently arrived migrants. On the other hand, utilization of YCs appears to improve over time. Conversely, the lower prevalence of YC utilization among those with temporary residence permits may suggest that they are unaware of their full entitlement to services on the same terms as those with permanent residence permits, in contrast to adult asylum seekers and undocumented migrants [20]. Research on SRH service utilization among adolescents and young people from migrant and refugee backgrounds in high-income settings reveals that, despite their diverse countries of origin and destination, these young migrants share remarkable similarities in their perceptions and experiences with SRH services [30]. Migrant status and the migration process significantly impact their awareness of care entitlements and service availability, regardless of their socioeconomic or legal status [43]. Additionally, awareness and utilization patterns may be influenced by unexamined factors or more complex interactions. Previous research underscores that the barriers faced by young migrants are complex and multifaceted, encompassing not just individual characteristics and knowledge gaps but also linguistic, cultural, and structural barriers, as well as discriminatory attitudes and policies. These intricate dynamics shape their perceptions and SRH-seeking behaviours [2,12,13,15,16,30,36].

## Strengths and limitations

This study has both strengths and limitations. First, to the best of our knowledge, this survey marks the first attempt to assess levels of awareness and utilization of YCs among migrants attending Swedish language programmes. By doing so, we significantly enrich the field with valuable insights into this insufficiently explored area. Second, our study draws from the mini-Sweden sample, which includes participants from 30 municipalities across the country. This stratified approach ensures representation from diverse regions and migrant populations. The sample spans from the northern municipality of Övertorneå to the southern municipality of Karlshamn. Third, the survey instrument was thoughtfully designed, drawing insights from existing literature, thereby enhancing the validity of our findings [2,14,29]. In addition, the questionnaire was accessible in six languages, enabling a significant number of migrants who were not fluent in Swedish to participate. The application of a Bayesian approach to regression analyses allows us largely to overcome the limitations inherent in relatively small sample sizes.

However, certain considerations must be taken into account when interpreting our findings. While cross-sectional studies provide valuable snapshots, they should not be used to infer causal relationships. We recommend caution when trying to generalize the results to all migrants since the study was conducted at language schools and introduction programmes and may thus mainly include recent migrants. Additionally, the low response rate for the awareness outcome may introduce selection bias, and the self-response nature of the study design could result in recall and response biases. The validity of our questionnaire remains uncertain: future studies could validate its effectiveness through comparison with other measures. It is important to consider that some participants completed the survey at home or in a classroom setting, which might have affected the privacy of their responses, especially if family members or classmates were present. Finally, although the survey was available in six languages, it may have inadvertently excluded individuals who do not speak these specific languages. However, it is worth noting that these languages align with the major migrant communities residing in Sweden.

Despite these limitations, this study makes a substantial contribution to our comprehension of migrants’ awareness and utilization of YCs. Moreover, it provides a solid foundation for future research to build upon these findings.

## Conclusion

This study highlights the level of awareness of YCs among migrants attending Swedish language programmes and their utilization by those aged 15−25 years, potentially impacting their access to available services and support. Descriptive and bivariate analyses reveal disparities, emphasizing the need for targeted interventions to address the specific needs of different socio-demographic groups and ensure equitable access to YCs’ information and services. The multivariate results underscore the role of time since migration in determining awareness and utilization of YCs, emphasizing the importance of sustainable strategies beyond one-time interventions. Further research is needed to investigate unexplored factors and complex interactions influencing awareness and utilization patterns. A deep understanding of factors underlying awareness and utilization of YCs will help identify effective strategies to improve young migrants’ access to crucial information and services.

## Data Availability

The datasets used and/or analyzed during the current study are not publicly available due to Swedish legal restrictions on personal data and constraints following ethical approval. Descriptive data may be provided in the format of plots, tables, or graphs by the corresponding author on reasonable request in accordance with the procedures specified in the ethical approval.
